# Supplementary material for: Epidemiology and nomogram of pediatric and young adulthood osteosarcoma patients with synchronous lung metastasis: A SEER analysis
Source: PLoS One. 2023 Jul 12;18(7):e0288492. doi: 10.1371/journal.pone.0288492 (PMC10337906; doi:10.1371/journal.pone.0288492)
Supplement: S1 Checklist — (DOCX) [file pone.0288492.s001.docx]

STROBE Statement—checklist of items that should be included in reports of observational studies

|  | Item No. | Recommendation | Page  No. | Relevant text from manuscript |
| --- | --- | --- | --- | --- |
| **Title and abstract** | 1 | (*a*) Indicate the study’s design with a commonly used term in the title or the abstract | Page 2 | a comprehensive analysis |
|  |  | (*b*) Provide in the abstract an informative and balanced summary of what was done and what was found | Page 2 | This study performed a comprehensive analysis regarding pediatric and young adulthood osteosarcoma patients had SLM. A visual, clinically operable, and easy-to-interpret nomogram model was developed for predicting the risk of SLM, which could be used in clinic and help clinicians make better decisions. |
| Introduction | | | |  |
| Background/rationale | 2 | Explain the scientific background and rationale for the investigation being reported | Page 3 | Currently, a few studies explored the risk factors of lung metastasis occurrence and constructed predictive models. However, no study focuses on the risk factors for pediatric and young adulthood patients. |
| Objectives | 3 | State specific objectives, including any prespecified hypotheses | Page 3 | this study aimed to describe the epidemiological trend of synchronous lung metastasis (SLM) at diagnosis in pediatric and young adulthood osteosarcoma patients, and identify risk factors and develop a clinically operable and easy-to-interpret nomogram model by using the big data from Surveillance, Epidemiology, and End Results (SEER) database. |
| Methods | | | |  |
| Study design | 4 | Present key elements of study design early in the paper | Page 4 | eFigure 1 |
| Setting | 5 | Describe the setting, locations, and relevant dates, including periods of recruitment, exposure, follow-up, and data collection | Page 4 | all data were extracted from SEER 17 registries  year 2010-2019 |
| Participants | 6 | (*a*) *Cohort study*—Give the eligibility criteria, and the sources and methods of selection of participants. Describe methods of follow-up  *Case-control study*—Give the eligibility criteria, and the sources and methods of case ascertainment and control selection. Give the rationale for the choice of cases and controls  *Cross-sectional study*—Give the eligibility criteria, and the sources and methods of selection of participants | Page 4 | The criteria used to identify eligible cases and the study design were presented in eFigure 1.  The following variables were extracted from the database |
|  |  | (*b*) *Cohort study*—For matched studies, give matching criteria and number of exposed and unexposed  *Case-control study*—For matched studies, give matching criteria and the number of controls per case |  |  |
| Variables | 7 | Clearly define all outcomes, exposures, predictors, potential confounders, and effect modifiers. Give diagnostic criteria, if applicable | Page 4-5 | age-standardized incidence rate  nomogram |
| Data sources/ measurement | 8* | For each variable of interest, give sources of data and details of methods of assessment (measurement). Describe comparability of assessment methods if there is more than one group | Page 4-5 | annual percentage change;  Univariate and multivariate logistic regression analyses |
| Bias | 9 | Describe any efforts to address potential sources of bias | NA |  |
| Study size | 10 | Explain how the study size was arrived at | NA |  |

Continued on next page

| Quantitative variables | 11 | Explain how quantitative variables were handled in the analyses. If applicable, describe which groupings were chosen and why | NA |  |
| --- | --- | --- | --- | --- |
| Statistical methods | 12 | (*a*) Describe all statistical methods, including those used to control for confounding | Page 5 | Other statistical analyses |
|  |  | (*b*) Describe any methods used to examine subgroups and interactions |  |  |
|  |  | (*c*) Explain how missing data were addressed | NA |  |
|  |  | (*d*) *Cohort study*—If applicable, explain how loss to follow-up was addressed  *Case-control study*—If applicable, explain how matching of cases and controls was addressed  *Cross-sectional study*—If applicable, describe analytical methods taking account of sampling strategy |  |  |
|  |  | (*e*) Describe any sensitivity analyses | NA |  |
| Results | | | | |
| Participants | 13* | (a) Report numbers of individuals at each stage of study—eg numbers potentially eligible, examined for eligibility, confirmed eligible, included in the study, completing follow-up, and analysed | Page 5 | A total of 1,965 pediatric and young adulthood osteosarcoma patients were identified. Among them, 278 out of 1965 patients presented with SLM at diagnosis, accounting for 14.1%. |
|  |  | (b) Give reasons for non-participation at each stage | NA |  |
|  |  | (c) Consider use of a flow diagram | NA |  |
| Descriptive data | 14* | (a) Give characteristics of study participants (eg demographic, clinical, social) and information on exposures and potential confounders | Page 5 | Table 1 |
|  |  | (b) Indicate number of participants with missing data for each variable of interest | NA |  |
|  |  | (c) *Cohort study*—Summarise follow-up time (eg, average and total amount) | NA |  |
| Outcome data | 15* | *Cohort study*—Report numbers of outcome events or summary measures over time | *NA* |  |
|  |  | *Case-control study—*Report numbers in each exposure category, or summary measures of exposure |  |  |
|  |  | *Cross-sectional study—*Report numbers of outcome events or summary measures |  |  |
| Main results | 16 | (*a*) Give unadjusted estimates and, if applicable, confounder-adjusted estimates and their precision (eg, 95% confidence interval). Make clear which confounders were adjusted for and why they were included | Page 5-6 |  |
|  |  | (*b*) Report category boundaries when continuous variables were categorized | *NA* |  |
|  |  | (*c*) If relevant, consider translating estimates of relative risk into absolute risk for a meaningful time period | *NA* |  |

Continued on next page

| Other analyses | 17 | Report other analyses done—eg analyses of subgroups and interactions, and sensitivity analyses | *NA* |  |
| --- | --- | --- | --- | --- |
| Discussion | | | | |
| Key results | 18 | Summarise key results with reference to study objectives | Page 7 | approximately 14.1% of all pediatric and young adulthood osteosarcoma patients developed a SLM at diagnosis. The rate was lower than that of entire osteosarcoma patients with SLM, mainly because of a relatively higher proportion of SLM in the elder patients |
| Limitations | 19 | Discuss limitations of the study, taking into account sources of potential bias or imprecision. Discuss both direction and magnitude of any potential bias | Page 9 | There are some limitations in this study. First, the nature of retrospective study might lead to data bias. Second, though we select patients from the SEER database, the sample size of this study is still limited because of the rare incidence of osteosarcoma. Besides, we did not perform external validation. Hence, more samples are warranted to identify the predictive power of the nomogram. Third, the information available from SEER database are limited, many associated risk factors mentioned above are not provided. Further studies should include more factors to construct a more powerful model. Last, limited to the follow-up time, the long-term survival for pediatric and young adulthood osteosarcoma patients with SLM could not be estimated accurately. This should be investigated in the future studies. |
| Interpretation | 20 | Give a cautious overall interpretation of results considering objectives, limitations, multiplicity of analyses, results from similar studies, and other relevant evidence | Page 7-8 |  |
| Generalisability | 21 | Discuss the generalisability (external validity) of the study results | NA |  |
| Other information | |  | | |
| Funding | 22 | Give the source of funding and the role of the funders for the present study and, if applicable, for the original study on which the present article is based | NA |  |

*Give information separately for cases and controls in case-control studies and, if applicable, for exposed and unexposed groups in cohort and cross-sectional studies.

**Note:** An Explanation and Elaboration article discusses each checklist item and gives methodological background and published examples of transparent reporting. The STROBE checklist is best used in conjunction with this article (freely available on the Web sites of PLoS Medicine at http://www.plosmedicine.org/, Annals of Internal Medicine at http://www.annals.org/, and Epidemiology at http://www.epidem.com/). Information on the STROBE Initiative is available at www.strobe-statement.org.
