# Supplementary material for: Epidemiology and nomogram of pediatric and young adulthood osteosarcoma patients with synchronous lung metastasis: A SEER analysis
Source: PLoS One. 2023 Jul 12;18(7):e0288492. doi: 10.1371/journal.pone.0288492 (PMC10337906; doi:10.1371/journal.pone.0288492)
Supplement: S1 Table — ASIR, age-standardized incidence rate; SEER, Surveillance, Epidemiology, and End Results. Rates are per 1,000,000 person-years. (DOCX) [file pone.0288492.s003.docx]

S1 Table: Age-standardized incidence rate of pediatric and young adulthood osteosarcoma patients, United States, 2010 to 2019.

|  | **Year** | **ASIR** | **N** | **SEER-17 population** |  | **Year** | **ASIR** | **N** | **SEER-17 population** |
| --- | --- | --- | --- | --- | --- | --- | --- | --- | --- |
| **All pediatric and young adulthood osteosarcoma patients** | 2010 | 3.31 | 152 | 45,481,932 | **Patients with synchronous lung metastasis** | 2010 | 0.46 | 21 | 45,481,932 |
|  | 2011 | 4.48 | 203 | 45,561,669 |  | 2011 | 0.54 | 24 | 45,561,669 |
|  | 2012 | 4.18 | 188 | 45,669,070 |  | 2012 | 0.65 | 29 | 45,669,070 |
|  | 2013 | 3.88 | 178 | 45,812,357 |  | 2013 | 0.55 | 25 | 45,812,357 |
|  | 2014 | 4.87 | 221 | 45,998,260 |  | 2014 | 0.60 | 27 | 45,998,260 |
|  | 2015 | 4.41 | 205 | 46,171,996 |  | 2015 | 0.62 | 29 | 46,171,996 |
|  | 2016 | 4.44 | 203 | 46,363,235 |  | 2016 | 0.72 | 33 | 46,363,235 |
|  | 2017 | 4.47 | 204 | 46,471,094 |  | 2017 | 0.60 | 27 | 46,471,094 |
|  | 2018 | 4.47 | 207 | 46,470,611 |  | 2018 | 0.73 | 33 | 46,470,611 |
|  | 2019 | 4.42 | 204 | 46,375,423 |  | 2019 | 0.66 | 30 | 46,375,423 |
| **Patients with synchronous lung metastasis/Male** | 2010 | 0.59 | 14 | 23,163,345 | **Patients with synchronous lung metastasis/Female** | 2010 | 0.32 | 7 | 22,318,587 |
|  | 2011 | 0.79 | 18 | 23,204,663 |  | 2011 | 0.27 | 6 | 22,357,006 |
|  | 2012 | 0.69 | 16 | 23,267,465 |  | 2012 | 0.61 | 13 | 22,401,605 |
|  | 2013 | 0.69 | 16 | 23,347,034 |  | 2013 | 0.40 | 9 | 22,465,323 |
|  | 2014 | 0.74 | 17 | 23,452,896 |  | 2014 | 0.47 | 10 | 22,545,364 |
|  | 2015 | 0.61 | 15 | 23,550,253 |  | 2015 | 0.62 | 14 | 22,621,743 |
|  | 2016 | 0.84 | 20 | 23,655,683 |  | 2016 | 0.59 | 13 | 22,707,552 |
|  | 2017 | 0.92 | 21 | 23,718,184 |  | 2017 | 0.27 | 6 | 22,752,910 |
|  | 2018 | 0.86 | 20 | 23,724,233 |  | 2018 | 0.60 | 13 | 22,746,378 |
|  | 2019 | 0.74 | 17 | 23,680,712 |  | 2019 | 0.58 | 13 | 22,694,711 |
| **Patients with synchronous lung metastasis/Age 1-9 years** | 2010 | 0.09 | 1 | 11,098,445 | **Patients with synchronous lung metastasis/Age 10-19 years** | 2010 | 1.05 | 12 | 11,618,482 |
|  | 2011 | 0.46 | 5 | 11,101,913 |  | 2011 | 1.04 | 12 | 11,510,785 |
|  | 2012 | 0.37 | 4 | 11,099,353 |  | 2012 | 1.68 | 19 | 11,400,346 |
|  | 2013 | 0.27 | 3 | 11,089,701 |  | 2013 | 1.40 | 16 | 11,325,967 |
|  | 2014 | 0.46 | 5 | 11,071,256 |  | 2014 | 1.78 | 20 | 11,275,508 |
|  | 2015 | 0.09 | 1 | 11,051,932 |  | 2015 | 1.51 | 17 | 11,252,051 |
|  | 2016 | 0.46 | 5 | 11,017,868 |  | 2016 | 1.77 | 20 | 11,253,711 |
|  | 2017 | 0.00 | 0 | 10,947,557 |  | 2017 | 1.60 | 18 | 11,272,532 |
|  | 2018 | 0.47 | 5 | 10,843,263 |  | 2018 | 2.04 | 23 | 11,281,061 |
|  | 2019 | 0.65 | 7 | 10,745,475 |  | 2019 | 1.33 | 15 | 11,245,710 |
| **Patients with synchronous lung metastasis/Age 20-39 years** | 2010 | 0.34 | 8 | 22,765,005 | **Patients with synchronous lung metastasis/White** | 2010 | 0.39 | 13 | 33,427,206 |
|  | 2011 | 0.32 | 7 | 22,948,971 |  | 2011 | 0.57 | 19 | 33,382,707 |
|  | 2012 | 0.26 | 6 | 23,169,371 |  | 2012 | 0.76 | 25 | 33,364,726 |
|  | 2013 | 0.25 | 6 | 23,396,689 |  | 2013 | 0.60 | 20 | 33,370,359 |
|  | 2014 | 0.08 | 2 | 23,651,496 |  | 2014 | 0.52 | 17 | 33,396,323 |
|  | 2015 | 0.42 | 11 | 23,868,013 |  | 2015 | 0.62 | 21 | 33,395,992 |
|  | 2016 | 0.31 | 8 | 24,091,656 |  | 2016 | 0.87 | 29 | 33,418,703 |
|  | 2017 | 0.38 | 9 | 24,251,005 |  | 2017 | 0.65 | 21 | 33,368,455 |
|  | 2018 | 0.20 | 5 | 24,346,287 |  | 2018 | 0.68 | 22 | 33,259,249 |
|  | 2019 | 0.32 | 8 | 24,384,238 |  | 2019 | 0.63 | 21 | 33,081,501 |
| **Patients with synchronous lung metastasis/Black** | 2010 | 0.60 | 4 | 6,132,882 | **Patients with synchronous lung metastasis/Other races** | 2010 | 0.73 | 4 | 5,921,844 |
|  | 2011 | 0.53 | 3 | 6,173,197 |  | 2011 | 0.37 | 2 | 6,005,765 |
|  | 2012 | 0.47 | 3 | 6,213,060 |  | 2012 | 0.19 | 1 | 6,091,284 |
|  | 2013 | 0.68 | 4 | 6,252,209 |  | 2013 | 0.18 | 1 | 6,189,789 |
|  | 2014 | 1.10 | 7 | 6,304,284 |  | 2014 | 0.54 | 3 | 6,297,653 |
|  | 2015 | 0.75 | 5 | 6,352,874 |  | 2015 | 0.50 | 3 | 6,423,130 |
|  | 2016 | 0.29 | 2 | 6,404,800 |  | 2016 | 0.35 | 2 | 6,539,732 |
|  | 2017 | 0.46 | 3 | 6,452,648 |  | 2017 | 0.52 | 3 | 6,649,991 |
|  | 2018 | 1.27 | 8 | 6,490,320 |  | 2018 | 0.49 | 3 | 6,721,042 |
|  | 2019 | 0.79 | 5 | 6,526,378 |  | 2019 | 0.66 | 4 | 6,767,544 |
| **Patients with synchronous lung metastasis/Appendicular** | 2010 | 0.41 | 19 | 45,481,932 | **Patients with synchronous lung metastasis/Axial** | 2010 | 0.04 | 2 | 45,481,932 |
|  | 2011 | 0.43 | 19 | 45,561,669 |  | 2011 | 0.11 | 5 | 45,561,669 |
|  | 2012 | 0.54 | 24 | 45,669,070 |  | 2012 | 0.11 | 5 | 45,669,070 |
|  | 2013 | 0.40 | 18 | 45,812,357 |  | 2013 | 0.15 | 7 | 45,812,357 |
|  | 2014 | 0.56 | 25 | 45,998,260 |  | 2014 | 0.05 | 2 | 45,998,260 |
|  | 2015 | 0.47 | 22 | 46,171,996 |  | 2015 | 0.14 | 7 | 46,171,996 |
|  | 2016 | 0.57 | 26 | 46,363,235 |  | 2016 | 0.14 | 7 | 46,363,235 |
|  | 2017 | 0.49 | 22 | 46,471,094 |  | 2017 | 0.11 | 5 | 46,471,094 |
|  | 2018 | 0.67 | 30 | 46,470,611 |  | 2018 | 0.07 | 3 | 46,470,611 |
|  | 2019 | 0.58 | 26 | 46,375,423 |  | 2019 | 0.08 | 4 | 46,375,423 |

ASIR, age-standardized incidence rate; SEER, Surveillance, Epidemiology, and End Results. Rates are per 1,000,000 person-years.
