# Supplementary material for: Epidemiology and nomogram of pediatric and young adulthood osteosarcoma patients with synchronous lung metastasis: A SEER analysis
Source: PLoS One. 2023 Jul 12;18(7):e0288492. doi: 10.1371/journal.pone.0288492 (PMC10337906; doi:10.1371/journal.pone.0288492)
Supplement: S2 Table — SSM, site-specific metastasis; SLM, synchronous lung metastasis. (DOCX) [file pone.0288492.s004.docx]

S2 Table: Baseline characteristics comparison between train and validation cohorts after propensity score matching.

| **Variable** | **Train cohort,**  **N=1,375 (%)** | **Validation cohort,**  **N=590 (%)** | ***P-value*** |
| --- | --- | --- | --- |
| **Age (years)** |  |  | *0.327* |
| 1-9 | 151 (11) | 52 (9) |  |
| 10-19 | 648 (47) | 290 (49) |  |
| 20-39 | 576 (42) | 248 (42) |  |
| **Race** |  |  | *0.880* |
| White | 1,043 (76) | 443 (75) |  |
| Black | 184 (13) | 84 (14) |  |
| Others | 148 (11) | 63 (11) |  |
| **Gender** |  |  | *0.181* |
| Male | 783 (57) | 316 (54) |  |
| Female | 592 (43) | 274 (46) |  |
| **Year of diagnosis** |  |  | *0.811* |
| 2010 | 102 (7) | 50 (8) |  |
| 2011 | 139 (10) | 64 (11) |  |
| 2012 | 141 (10) | 47 (8) |  |
| 2013 | 123 (9) | 55 (9) |  |
| 2014 | 148 (11) | 73 (12) |  |
| 2015 | 140 (10) | 65 (11) |  |
| 2016 | 146 (11) | 57 (10) |  |
| 2017 | 145 (11) | 59 (10) |  |
| 2018 | 149 (11) | 58 (10) |  |
| 2019 | 142 (10) | 62 (11) |  |
| **Primary site** |  |  | *0.305* |
| Appendicula | 1,077 (78) | 449 (76) |  |
| Axial | 298 (22) | 141 (24) |  |
| **Tumor grade** |  |  | *0.252* |
| Low grade | 774 (56) | 326 (55) |  |
| High grade | 281 (20) | 139 (24) |  |
| Unknown | 320 (23) | 125 (21) |  |
| **Tumor size (cm)** |  |  | *0.829* |
| < 5 | 206 (15) | 92 (16) |  |
| 5-10 | 486 (35) | 198 (34) |  |
| ≥ 10 | 503 (37) | 216 (37) |  |
| Unknown | 180 (13) | 84 (14) |  |
| **Lymph node status** |  |  | *0.389* |
| Negative | 1,242 (90) | 539 (91) |  |
| Positive | 28 (2) | 15 (3) |  |
| Unknown | 105 (8) | 36 (6) |  |
| **Other SSM** |  |  | *1.000* |
| No | 1,324 (96) | 568 (96) |  |
| Yes | 51 (4) | 22 (4) |  |
| **SLM** |  |  | *0.399* |
| No | 1,174 (85) | 513 (87) |  |
| Yes | 201 (15) | 77 (13) |  |
| SSM, site-specific metastasis; SLM, synchronous lung metastasis. | | | |
