# Supplementary material for: Epidemiology and nomogram of pediatric and young adulthood osteosarcoma patients with synchronous lung metastasis: A SEER analysis
Source: PLoS One. 2023 Jul 12;18(7):e0288492. doi: 10.1371/journal.pone.0288492 (PMC10337906; doi:10.1371/journal.pone.0288492)
Supplement: S3 Table — OR, odds ratio; CI, confidence interval; SSM, site-specific metastasis. (DOCX) [file pone.0288492.s005.docx]

S3 Table: Risk factors associated with synchronous lung metastasis in pediatric and young adulthood osteosarcoma patients.

| **Variable** | **Univariate** | | **Multivariate** | |
| --- | --- | --- | --- | --- |
|  | **OR (95% CI)** | ***P-value*** | **OR (95% CI)** | ***P-value*** |
| **Age (years)** |  |  |  |  |
| 1-9 | Reference | - | Reference | - |
| 10-19 | 1.20 (0.76-1.97) | *0.439* | 1.20 (0.73-2.07) | *0.484* |
| 20-39 | 0.49 (0.29-0.83) | *0.007* | 0.67 (0.37-1.22) | *0.182* |
| **Race** |  |  |  |  |
| White | Reference | - |  |  |
| Black | 1.12 (0.72-1.70) | *0.590* |  |  |
| Others | 0.75 (0.43-1.24) | *0.289* |  |  |
| **Gender** |  |  |  |  |
| Female | Reference | - | Reference | - |
| Male | 1.46 (1.07-2.00) | *0.017* | 1.31 (0.93-1.86) | *0.120* |
| **Primary site** |  |  |  |  |
| Appendicular | Reference | - | Reference | - |
| Axial | 0.65 (0.43-0.95) | *0.033* | 1.18 (0.72-1.89) | *0.511* |
| **Tumor grade** |  |  |  |  |
| High grade | Reference | - | Reference | - |
| Low grade | 0.12 (0.06-0.24) | *< 0.001* | 0.21 (0.09-0.44) | *< 0.001* |
| Unknown | 0.69 (0.48-0.99) | *0.047* | 0.66 (0.43-0.98) | *0.045* |
| **Tumor size (cm)** |  |  |  |  |
| < 5 | Reference | - | Reference | - |
| 5-10 | 2.18 (1.09-4.85) | *0.038* | 1.13 (0.55-2.59) | *0.748* |
| ≥ 10 | 6.78 (3.56-14.65) | *< 0.001* | 3.37 (1.71-7.47) | *0.001* |
| Unknown | 4.20 (2.01-9.67) | *< 0.001* | 2.28 (1.02-5.49) | *0.052* |
| **Lymph node status** |  |  |  |  |
| Negative | Reference | - | Reference | - |
| Positive | 7.33 (3.42-15.90) | *< 0.001* | 5.64 (2.40-13.26) | *< 0.001* |
| Unknown | 1.23 (0.69-2.06) | *0.462* | 0.96 (0.50-1.77) | *0.908* |
| **Other SSM** |  |  |  |  |
| No | Reference | - | Reference | - |
| Yes | 15.26 (8.40-28.89) | *< 0.001* | 12.83 (6.68-25.69) | *< 0.001* |
| OR, odds ratio; CI, confidence interval; SSM, site-specific metastasis. | | | | |
