# Supplementary material for: Epidemiology and nomogram of pediatric and young adulthood osteosarcoma patients with synchronous lung metastasis: A SEER analysis
Source: PLoS One. 2023 Jul 12;18(7):e0288492. doi: 10.1371/journal.pone.0288492 (PMC10337906; doi:10.1371/journal.pone.0288492)
Supplement: S4 Table — SSM, site-specific metastasis. (DOCX) [file pone.0288492.s006.docx]

S4 Table: Nomogram point of each variable.

| **Variable** | **Point** |
| --- | --- |
| **Tumor grade** |  |
| High grade | 68 |
| Low grade | 0 |
| Unknown | 52 |
| **Tumor size (cm)** |  |
| < 5 | 0 |
| 5-10 | 7 |
| ≥ 10 | 51 |
| Unknown | 35 |
| **Lymph node status** |  |
| Negative | 1 |
| Positive | 61 |
| Unknown | 0 |
| **Other SSM** |  |
| No | 0 |
| Yes | 100 |

SSM, site-specific metastasis
