# Supplementary material for: Epidemiology and nomogram of pediatric and young adulthood osteosarcoma patients with synchronous lung metastasis: A SEER analysis
Source: PLoS One. 2023 Jul 12;18(7):e0288492. doi: 10.1371/journal.pone.0288492 (PMC10337906; doi:10.1371/journal.pone.0288492)
Supplement: S5 Table — HR, hazard ratio; CI, confidence interval; SSM, site-specific metastasis. (DOCX) [file pone.0288492.s007.docx]

S5 Table: Prognostic factors associated with cancer-specific survival in pediatric and young adulthood osteosarcoma patients with synchronous lung metastasis.

| **Variable** | **Multivariate** | |
| --- | --- | --- |
|  | **HR (95% CI)** | ***P-value*** |
| **Age (years)** |  |  |
| 1-9 | Reference | - |
| 10-19 | 1.68 (0.93-3.03) | *0.086* |
| 20-39 | 2.11 (1.10-4.02) | *0.024* |
| **Race** |  |  |
| White | Reference | - |
| Black | 2.20 (1.43-3.40) | *< 0.001* |
| Others | 0.67 (0.34-1.32) | *0.246* |
| **Gender** |  |  |
| Female | Reference | - |
| Male | 1.44 (1.00-2.07) | *0.050* |
| **Primary site** |  |  |
| Appendicular | Reference | - |
| Axial | 0.73 (0.43-1.24) | *0.241* |
| **Tumor grade** |  |  |
| High grade | Reference | - |
| Low grade | 0.47 (0.17-1.28) | *0.140* |
| Unknown | 0.61 (0.40-0.93) | *0.022* |
| **Tumor size (cm)** |  |  |
| < 5 | Reference | - |
| 5-10 | 1.01 (0.38-2.69) | *0.984* |
| ≥ 10 | 1.17 (0.46-2.98) | *0.738* |
| Unknown | 0.58 (0.20-1.62) | *0.296* |
| **Lymph node status** |  |  |
| Negative | Reference | - |
| Positive | 3.27 (1.90-5.63) | *< 0.001* |
| Unknown | 1.47 (0.80-2.70) | *0.218* |
| **Other SSM** |  |  |
| No | Reference | - |
| Yes | 1.96 (1.25-3.08) | *0.003* |
| **Surgery** |  |  |
| No | Reference | - |
| Yes | 0.22 (0.14-0.35) | *< 0.001* |
| **Radiotherapy** |  |  |
| No | Reference | - |
| Yes | 0.44 (0.16-1.23) | *0.119* |
| **Systemic treatment** |  |  |
| No | Reference | - |
| Yes | 1.01 (0.59-1.75) | *0.951* |
| HR, hazard ratio; CI, confidence interval; SSM, site-specific metastasis. | | |
