# Supplementary material for: Epidemiology and nomogram of pediatric and young adulthood osteosarcoma patients with synchronous lung metastasis: A SEER analysis
Source: PLoS One. 2023 Jul 12;18(7):e0288492. doi: 10.1371/journal.pone.0288492 (PMC10337906; doi:10.1371/journal.pone.0288492)
Supplement: S6 Table — (DOCX) [file pone.0288492.s008.docx]

S6 Table: Summary of targeted molecular and related inhibitor associated with osteosarcoma metastasis.

| Targeted molecular | Inhibitor or potential | Reference |
| --- | --- | --- |
| MDM2 | SAR405838 | [21] |
| VEGFR/PDGFR | Sunitinib  Pazopanib  Anlotinib  Sorafenib | [22] |
| mTOR | Rapamycin | [23] |
| IGF-1R | Cixutumumab | [24] |
| CDK | Flavopiridol | [25] |
| Aurora-B | VX-680  ZM447439  Alisertib | [26] |
| TP53 | AZD1775  ZN-c3 | [27] |
| MYC | 10058-F4 | [28] |
