# Supplementary material for: Epidemiology and nomogram of pediatric and young adulthood osteosarcoma patients with synchronous lung metastasis: A SEER analysis
Source: PLoS One. 2023 Jul 12;18(7):e0288492. doi: 10.1371/journal.pone.0288492 (PMC10337906; doi:10.1371/journal.pone.0288492)
Supplement: Attachment — Submitted filename: Author Response Letter.docx [file pone.0288492.s009.docx]

Dear Editor,

Thank you very much for your e-mail on 20 JUN 2023 to informing us to revise our manuscript (PONE-D-23-15475) and respond to the reviewer(s)' comments.

Here, we have carefully studied the reviewers’ comments and made the appropriate revisions to our manuscript.

The following are our specific responses to the editor’s and reviewers’ comments:

1. Please ensure that your manuscript meets PLOS ONE's style requirements, including those for file naming. The PLOS ONE style templates can be found at 
https://journals.plos.org/plosone/s/file?id=wjVg/PLOSOne_formatting_sample_main_body.pdf and https://journals.plos.org/plosone/s/file?id=ba62/PLOSOne_formatting_sample_title_authors_affiliations.pdf

Author response: Thanks for the editor’s comment. We have amended the style according to the requirements.

2. We note that you have indicated that data from this study are available upon request. PLOS only allows data to be available upon request if there are legal or ethical restrictions on sharing data publicly. For more information on unacceptable data access restrictions, please see http://journals.plos.org/plosone/s/data-availability#loc-unacceptable-data-access-restrictions. In your revised cover letter, please address the following prompts:

a) If there are ethical or legal restrictions on sharing a de-identified data set, please explain them in detail (e.g., data contain potentially sensitive information, data are owned by a third-party organization, etc.) and who has imposed them (e.g., an ethics committee). Please also provide contact information for a data access committee, ethics committee, or other institutional body to which data requests may be sent.

b) If there are no restrictions, please upload the minimal anonymized data set necessary to replicate your study findings as either Supporting Information files or to a stable, public repository and provide us with the relevant URLs, DOIs, or accession numbers. For a list of acceptable repositories, please see http://journals.plos.org/plosone/s/data-availability#loc-recommended-repositories.

We will update your Data Availability statement on your behalf to reflect the information you provide.

Author response: Thanks for the editor’s comment. All the data used in this study were obtained form the public database, SEER database, which is open for all researchers. We have provided sufficient patients selection criteria. Hence, we think there is no need to upload the minimal anonymized data set to replicate our study findings. Because they could replicate this study by access the SEER database for original data, which is more convincing. If needed, we could provide all the datasets used in this study upon reasonable request, by inquiring the corresponding author.

3. PLOS requires an ORCID iD for the corresponding author in Editorial Manager on papers submitted after December 6th, 2016. Please ensure that you have an ORCID iD and that it is validated in Editorial Manager. To do this, go to ‘Update my Information’ (in the upper left-hand corner of the main menu), and click on the Fetch/Validate link next to the ORCID field. This will take you to the ORCID site and allow you to create a new iD or authenticate a pre-existing iD in Editorial Manager. Please see the following video for instructions on linking an ORCID iD to your Editorial Manager account: <https://www.youtube.com/watch?v=_xcclfuvtxQ>

Author response: Thanks for the editor’s comment. Revised as required.

4. Please include your tables as part of your main manuscript and remove the individual files. Please note that supplementary tables (should remain/ be uploaded) as separate "supporting information" files

Author response: Thanks for the editor’s comment. Revised as required.

5. Please include captions for your Supporting Information files at the end of your manuscript, and update any in-text citations to match accordingly. Please see our Supporting Information guidelines for more information: http://journals.plos.org/plosone/s/supporting-information.

Author response: Thanks for the editor’s comment. Revised as required.

6. Please review your reference list to ensure that it is complete and correct. If you have cited papers that have been retracted, please include the rationale for doing so in the manuscript text, or remove these references and replace them with relevant current references. Any changes to the reference list should be mentioned in the rebuttal letter that accompanies your revised manuscript. If you need to cite a retracted article, indicate the article’s retracted status in the References list and also include a citation and full reference for the retraction notice.
Author response: Thanks for the editor’s comment. We have added some references in the discussion section. We have reviewed our reference list and ensure that it is complete and correct.

Reviewer #1: Minor revision
1. Since the growth and development of osteosarcoma depend on a tightly regulated and balanced angiogenesis and the blocking of tumor angiogenesis could be a promising strategy for cancer therapy, it is necessary to report in the Introduction some targets, which have already been identified.
An example is represented by cyclin-dependent kinases, fundamental mediators of neoangiogenesis and other (see de Nigris F, et al. Osteosarcoma cells induces endothelial cell proliferation during neo-angiogenesis. J Cell Physiol. 2013 Apr;228(4):846-52).

Author response: Thanks for the review’s comment. We have added this reference (ref. 20) in the **Discussion** section, paragraph 4, “Nigris and colleagues demonstrated that the growth and development of osteosarcoma, as well as the metastasis development, depend on a tightly regulated and balanced angiogenesis[20]. The blocking of tumor angiogenesis could be a promising strategy for osteosarcoma therapy.”

2. To organize a descriptive table of molecular targets, which are altered in the presence of osteosarcoma metastases.

Author response: Thanks for the review’s comment. We have added some discussion about the potential molecular targets in the presence of osteosarcoma metastases. And summarize them in **eTable 6**. See details in paragraph 4 of the **Discussion,** “In addition to that, multiple molecular regulation mechanisms may get involved in the metastasis of osteosarcoma. Studies demonstrated that MDM2 amplification[21], overexpression of VEGFR and PDGFR[22], the activation of mTOR[23], increase of IGF-1R[24], cyclin-dependent kinase[25], Aurora-B[26], TP53[27] and MYC[28] were associated with the metastasis of osteosarcoma. And some inhibitors or potential inhibitors could suppress the metastasis of osteosarcoma, then extend the survival (eTable 6). In addition, some molecules may also be involved in metachronous lung metastases, such as HER-2 expression[29], circFIRRE[30], CXCL1[31], and extracellular vesicles miR-101[32]. As lung metastasis is the most common involved organ at diagnosis, these factors could help for identifying SLM, and are worthwhile candidates for developing potential treatment regimen.”

Reviewer #2: Manuscript titled "Epidemiology and nomogram of pediatric and young adulthood osteosarcoma patients with synchronous lung metastasis: A SEER analysis" is a very interesting analysis of SLM risk in patients with osteosarcoma. The overall structure of the manuscript is of good quality, figures are clear and easy to understand. Methods and results are of good quality and support the initial hypothesis. Authors should improve the manuscript in some parts:
1. authors should add information on the pathophysiology of SLM events in these patients and the involvement of serum cytokines like IL-1, IL-6 and CXCL-12 in cancer metastasis as well as in cardiovascular side events.
Author response: Thanks for the review’s comment. We add some discussion about the involved cytokines in the paragraph 5 of **Discussion** section, “Some inflammatory cytokines may also get involved in the tumor progression, recurrence and metastasis of osteosarcoma[34]. A few studies demonstrated that the expression of IL-6, IL-8, and IL-1β could promote the tumor relapse and metastasis[35,36]. And drugs targeting IL-1 may be a potential method to improve the survival of metastatic osteosarcoma[37-40].”

2. In discussion, authors should add some data on the incidence of tumour-related and chemotherapy-related cardiovascular side events in these patients ( long term toxicity).
Author response: Thanks for the review’s comment. We add some discussion about the cardiovascular side events in the paragraph 7 of **Discussion** section, “Cardiovascular side event was a common and fatal event, which was demonstrated to be the leading cause of non-cancer death among osteosarcoma patients[43]. For osteosarcoma patients, the most often reason for inducing cardiovascular death was chemotherapy[44]. Other studies suggested that radiation and targeted therapy could also induce cardiovascular toxicity and affect survival[45,46]. More importantly, a few studies reported that the osteosarcoma could directly invade the cardiovascular system and cause damage, with an incidence of approximately 2%, but it increased into 20% when performed autopsy[47-49]”.

We hope that we have adequately addressed the constructive comments. We greatly appreciate your consideration of this manuscript for possible publication in your journal and look forward to hearing from you soon.

Sincerely,

Dr. Jin Yang
